# Supplementary material for: First-Trimester Screening for Miscarriage or Stillbirth—Prediction Model Based on MicroRNA Biomarkers
Source: Int J Mol Sci. 2023 Jun 14;24(12):10137. doi: 10.3390/ijms241210137 (PMC10299132; doi:10.3390/ijms241210137)
Supplement: Supplementary file 1 [file ijms-24-10137-s001.zip › Supplementary Figure S1.pdf]

Supplementary Figure S1.

A

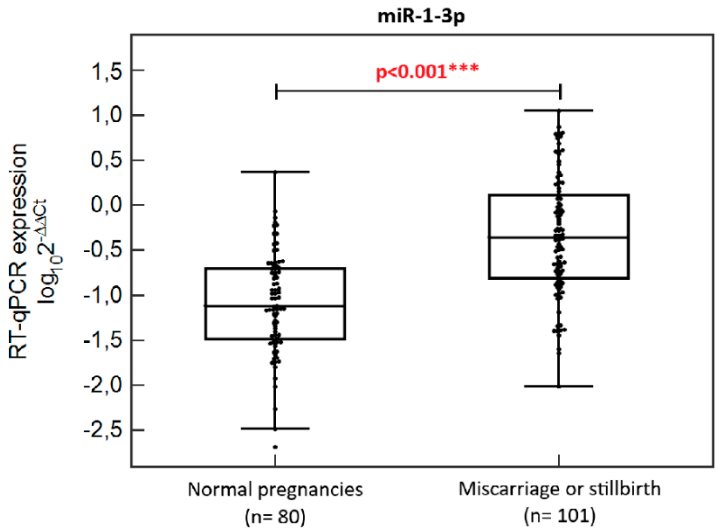

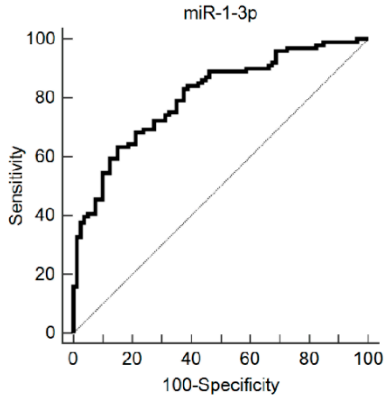

Area under the ROC curve (AUC)	0.806
Standard Error	0.0318
95% Confidence interval	0.741 to 0.861
Significance level P (Area=0.5)	<0.0001

Estimated sensitivity at fixed specificity			
Specificity	Sensitivity	95% CI	Criterion
90.00	54.46	38.61 to 72.28	>0.377841

Criterion	Sensitivity	95% CI	Specificity	95% CI	+LR	95% CI	-LR	95% CI
>0.23812	63.37	53.2 - 72.7	85.00	75.3 - 92.0	4.22	2.5 - 7.3	0.43	0.3 - 0.6

B

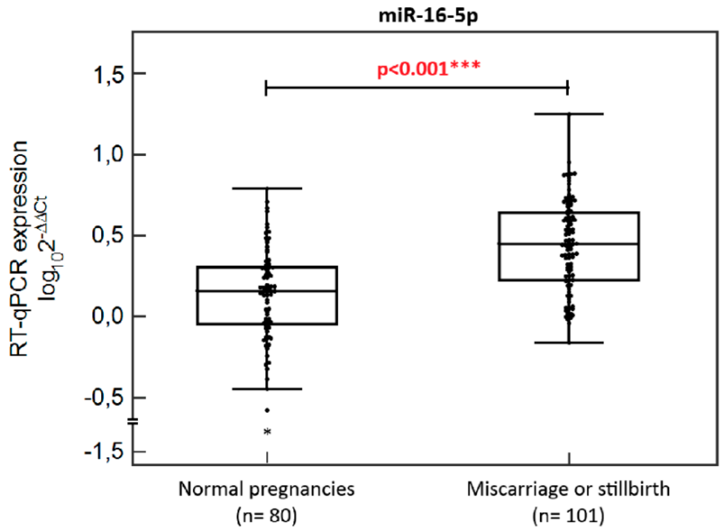

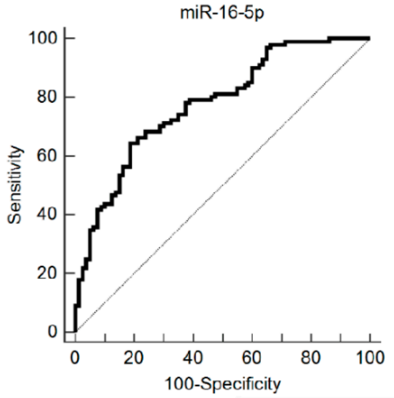

Area under the ROC curve (AUC)	0.777
Standard Error	0.0342
95% Confidence interval	0.709 to 0.835
Significance level P (Area=0.5)	<0.0001

Estimated sensitivity at fixed specificity			
Specificity	Sensitivity	95% CI	Criterion
90.00	43.56	25.74 to 62.38	>3.02212

Criterion	Sensitivity	95% CI	Specificity	95% CI	+LR	95% CI	-LR	95% CI
>2.212571	64.36	54.2 - 73.6	81.25	71.0 - 89.1	3.43	2.1 - 5.5	0.44	0.3 - 0.6

C

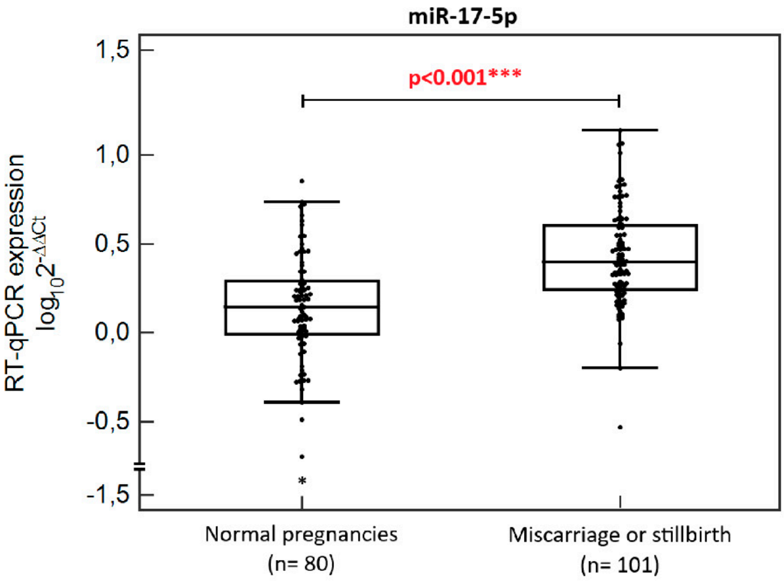

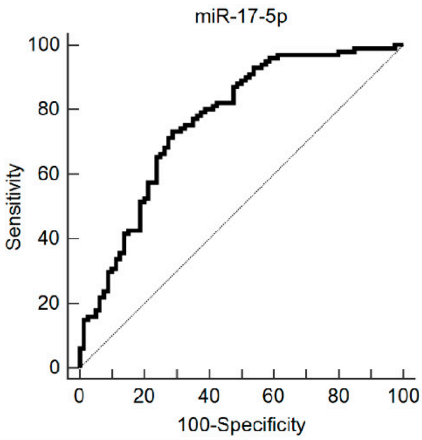

Area under the ROC curve (AUC)	0.766
Standard Error	0.0362
95% Confidence interval	0.698 to 0.826
Significance level P (Area=0.5)	<0.0001

Estimated sensitivity at fixed specificity			
Specificity	Sensitivity	95% CI	Criterion
90.00	30.69	13.86 to 48.51	>3.458809

Criterion	Sensitivity	95% CI	Specificity	95% CI	+LR	95% CI	-LR	95% CI
>1.780378	73.27	63.5 - 81.6	71.25	60.0 - 80.8	2.55	1.8 - 3.7	0.38	0.3 - 0.5

D

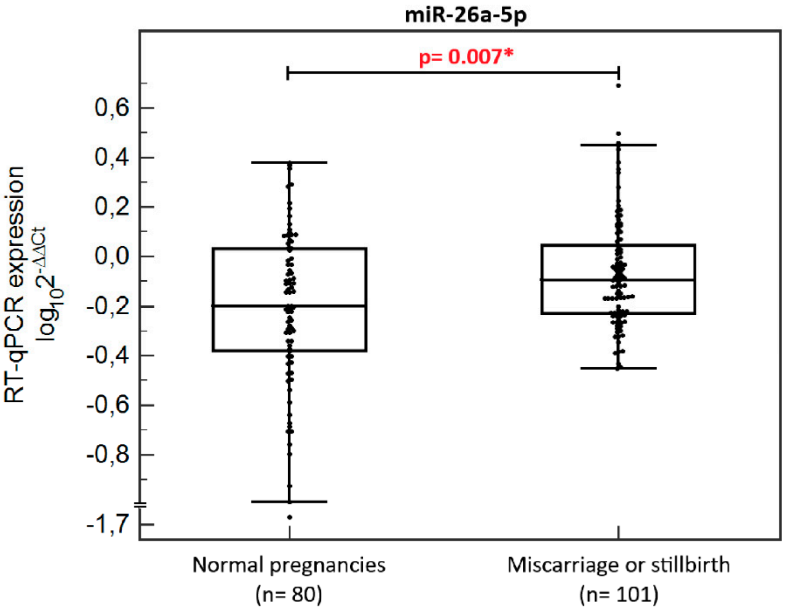

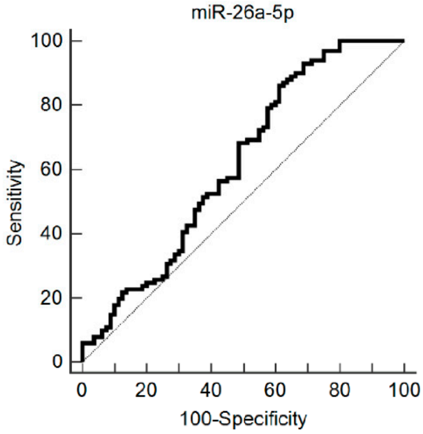

Area under the ROC curve (AUC)	0.617
Standard Error	0.0432
95% Confidence interval	0.542 to 0.688
Significance level P (Area=0.5)	0.0068

Estimated sensitivity at fixed specificity			
Specificity	Sensitivity	95% CI	Criterion
90.00	17.82	6.93 to 28.76	>1.350228

Criterion	Sensitivity	95% CI	Specificity	95% CI	+LR	95% CI	-LR	95% CI
>0.525138	86.14	77.8 - 92.2	38.75	28.1 - 50.3	1.41	1.2 - 1.7	0.36	0.2 - 0.6

E

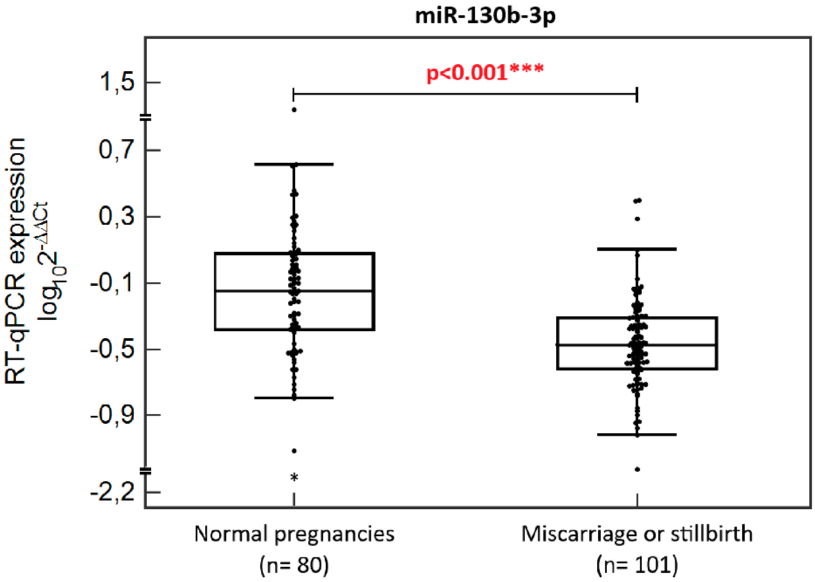

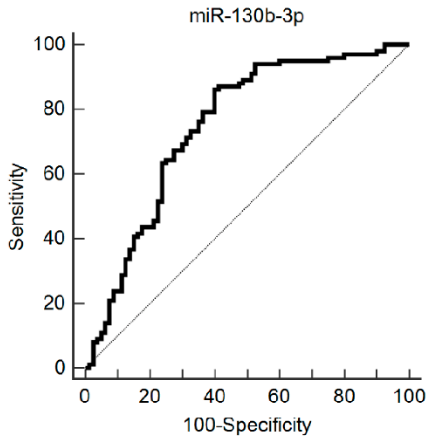

Area under the ROC curve (AUC)	0.749
Standard Error	0.0383
95% Confidence interval	0.679 to 0.810
Significance level P (Area=0.5)	<0.0001

Estimated sensitivity at fixed specificity

Specificity	Sensitivity	95% CI	Criterion
90.00	23.76	6.93 to 42.35	≤0.235772

Criterion	Sensitivity	95% CI	Specificity	95% CI	+LR	95% CI	-LR	95% CI
≤0.591670738	86.14	77.8 - 92.2	60.00	48.4 - 70.8	2.15	1.6 - 2.8	0.23	0.1 - 0.4

F

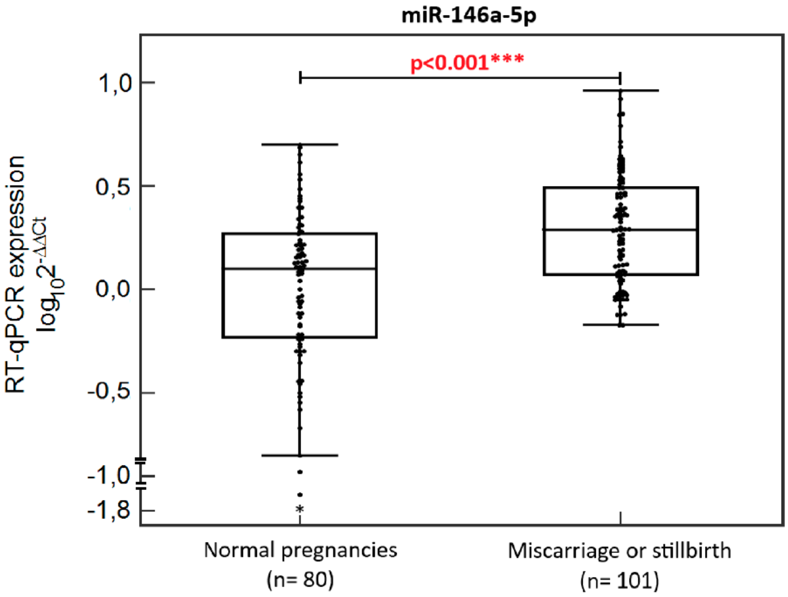

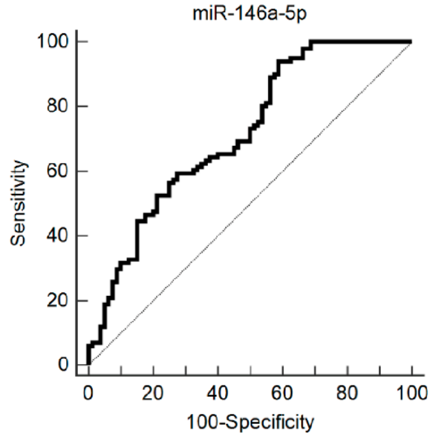

Area under the ROC curve (AUC)	0.717
Standard Error	0.0385
95% Confidence interval	0.645 to 0.781
Significance level P (Area=0.5)	<0.0001

Estimated sensitivity at fixed specificity

Specificity	Sensitivity	95% CI	Criterion
90.00	31.68	16.83 to 52.48	>2.744534

Criterion	Sensitivity	95% CI	Specificity	95% CI	+LR	95% CI	-LR	95% CI
>0.879161	94.06	87.5 - 97.8	41.25	30.4 - 52.8	1.60	1.3 - 1.9	0.14	0.06 - 0.3

G

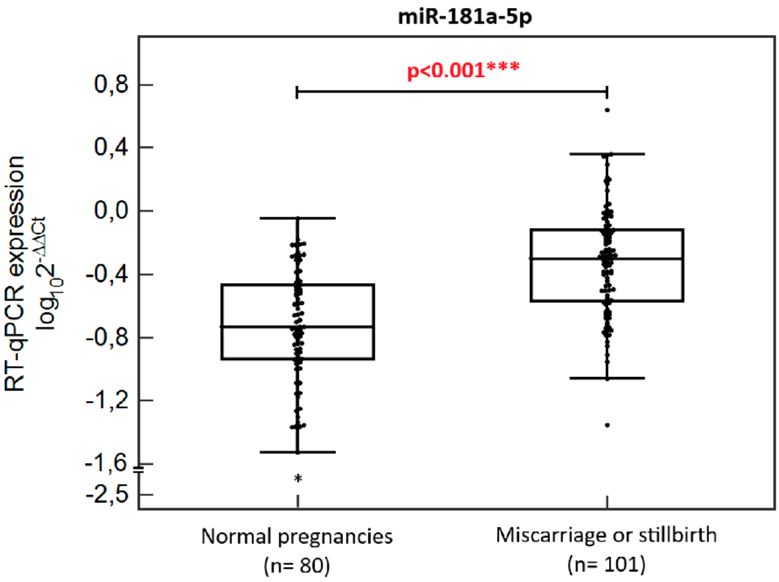

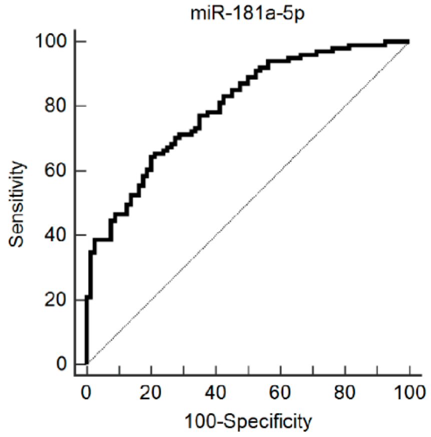

Area under the ROC curve (AUC)	0.799
Standard Error	0.0321
95% Confidence interval	0.734 to 0.855
Significance level P (Area=0.5)	<0.0001

Estimated sensitivity at fixed specificity			
Specificity	Sensitivity	95% CI	Criterion
90.00	46.53	32.67 to 61.52	>0.521629

Criterion	Sensitivity	95% CI	Specificity	95% CI	+LR	95% CI	-LR	95% CI
>0.368016	64.36	54.2 - 73.6	80.00	69.6 - 88.1	3.22	2.0 - 5.1	0.45	0.3 - 0.6

H

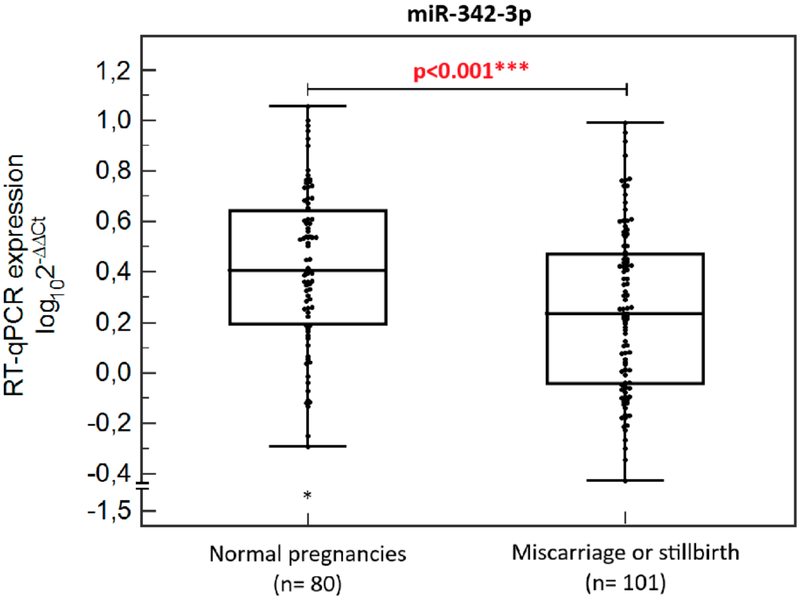

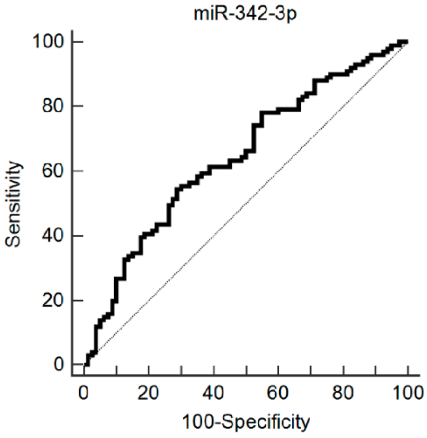

Area under the ROC curve (AUC)	0.645
Standard Error	0.0412
95% Confidence interval	0.571 to 0.715
Significance level P (Area=0.5)	0.0004

Estimated sensitivity at fixed specificity			
Specificity	Sensitivity	95% CI	Criterion
90.00	26.73	11.84 to 45.54	≤0.909153316

Criterion	Sensitivity	95% CI	Specificity	95% CI	+LR	95% CI	-LR	95% CI
≤1.797163562	54.46	44.2 - 64.4	71.25	60.0 - 80.8	1.89	1.3 - 2.8	0.64	0.5 - 0.8

I

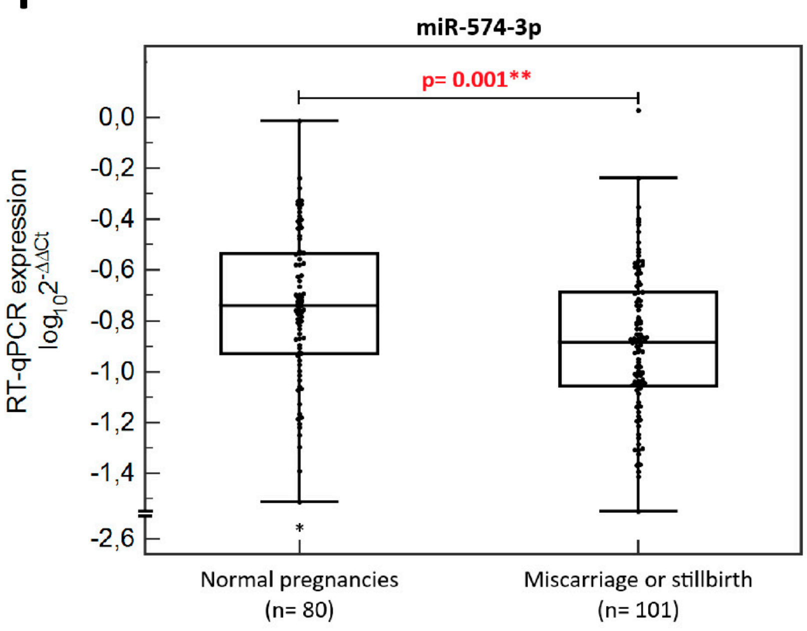

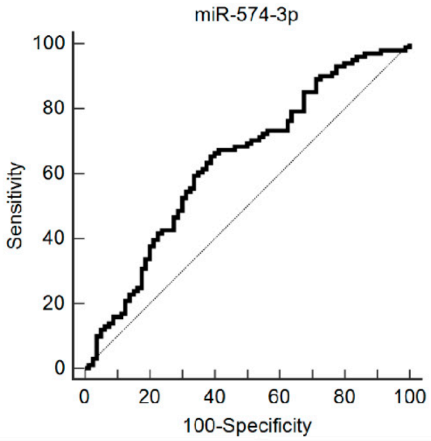

Area under the ROC curve (AUC)	0.636
Standard Error	0.0419
95% Confidence interval	0.561 to 0.706
Significance level P (Area=0.5)	0.0012

Estimated sensitivity at fixed specificity			
Specificity	Sensitivity	95% CI	Criterion
90.00	15.84	6.93 to 28.71	≤0.06522

Criterion	Sensitivity	95% CI	Specificity	95% CI	+LR	95% CI	-LR	95% CI
≤0.158363743	65.35	55.2 - 74.5	61.25	49.7 - 71.9	1.69	1.2 - 2.3	0.57	0.4 - 0.8

Supplementary Figure S1. Up-regulation of miR-1-3p (**A**), miR-16-5p (**B**), miR-17-5p (**C**), miR-26a-5p (**D**), miR-146a-5p (**F**), and miR-181a-5p (**G**) and down-regulation **of** miR-130b-3p (**E**), miR-342-3p (**H**), and miR-574-3p (**I**) differentiates in early stages of gestation between normal term pregnancies and pregnancies with subsequent miscarriage or stillbirth.

(results after the Benjamini-Hochberg correction are marked by * for α=0.05, ** for α=0.01, and *** for α=0.001)
