# Supplementary material for: First-Trimester Screening for Miscarriage or Stillbirth—Prediction Model Based on MicroRNA Biomarkers
Source: Int J Mol Sci. 2023 Jun 14;24(12):10137. doi: 10.3390/ijms241210137 (PMC10299132; doi:10.3390/ijms241210137)
Supplement: Supplementary file 1 [file ijms-24-10137-s001.zip › Supplementary Figure S2.pdf]

Supplementary Figure S2.

A

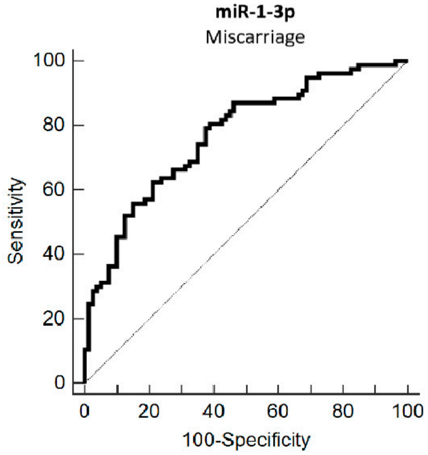

Area under the ROC curve (AUC)	0.770
Standard Error	0.0373
95% Confidence interval	0.696 to 0.833
Significance level P (Area=0.5)	<0.0001

Estimated sensitivity at fixed specificity			
Specificity	Sensitivity	95% CI	Criterion
90.00	45.45	25.97 to 63.64	>0.377841

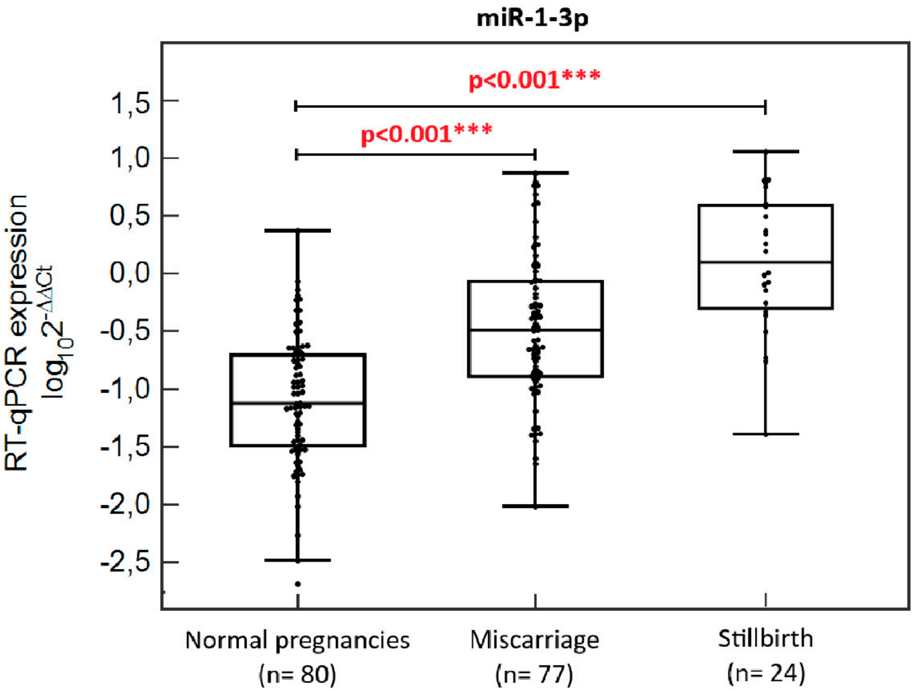

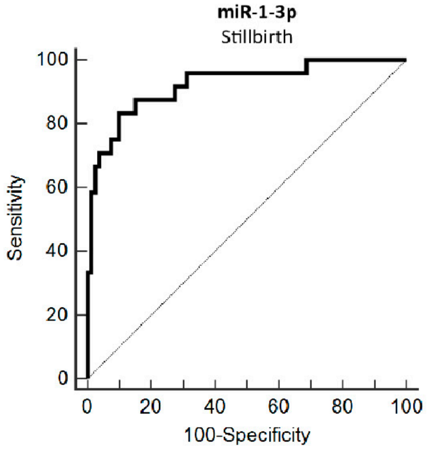

Area under the ROC curve (AUC)	0.922
Standard Error	0.0340
95% Confidence interval	0.853 to 0.966
Significance level P (Area=0.5)	<0.0001

Estimated sensitivity at fixed specificity			
Specificity	Sensitivity	95% CI	Criterion
90.00	83.33	62.50 to 97.39	>0.377841

Criterion	Sensitivity	95% CI	Specificity	95% CI	+LR	95% CI	-LR	95% CI
>0.115119	80.52	69.9 - 88.7	61.25	49.7 - 71.9	2.08	1.5 - 2.8	0.32	0.2 - 0.5

Criterion	Sensitivity	95% CI	Specificity	95% CI	+LR	95% CI	-LR	95% CI
>0.377841	83.33	62.6 - 95.3	90.00	81.2 - 95.6	8.33	4.2 - 16.5	0.19	0.08 - 0.5

B

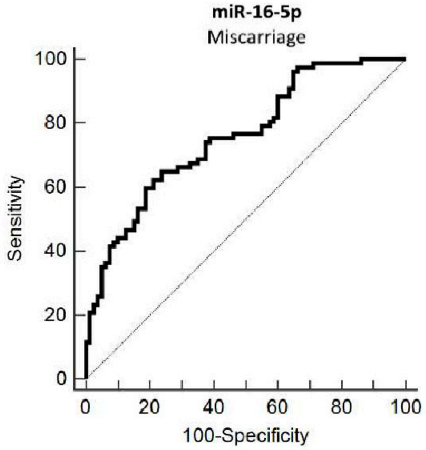

Area under the ROC curve (AUC)	0.757
Standard Error	0.0378
95% Confidence interval	0.683 to 0.822
Significance level P (Area=0.5)	<0.0001

Estimated sensitivity at fixed specificity			
Specificity	Sensitivity	95% CI	Criterion
90.00	44.16	28.57 to 59.74	>3.02212

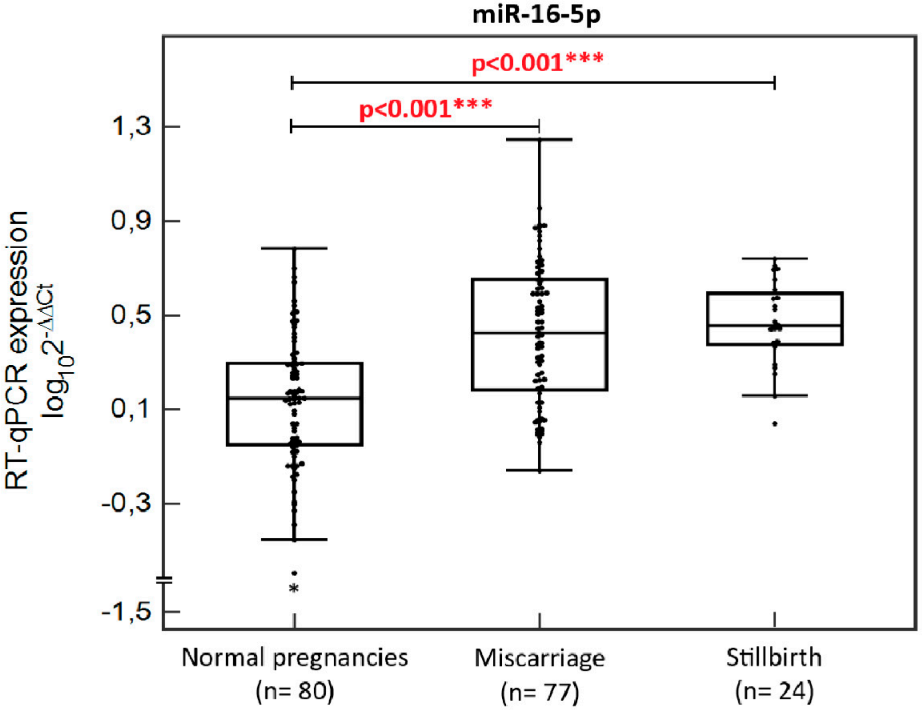

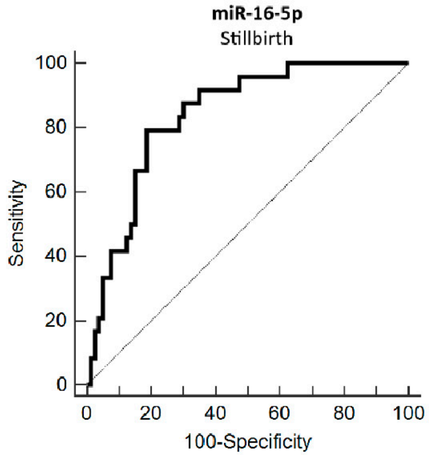

Area under the ROC curve (AUC)	0.839
Standard Error	0.0415
95% Confidence interval	0.754 to 0.903
Significance level P (Area=0.5)	<0.0001

Estimated sensitivity at fixed specificity			
Specificity	Sensitivity	95% CI	Criterion
90.00	41.67	16.67 to 75.00	>3.02212

Criterion	Sensitivity	95% CI	Specificity	95% CI	+LR	95% CI	-LR	95% CI
>1.983489	64.94	53.2 - 75.5	76.25	65.4 - 85.1	2.73	1.8 - 4.2	0.46	0.3 - 0.6

Criterion	Sensitivity	95% CI	Specificity	95% CI	+LR	95% CI	-LR	95% CI
>2.212571	79.17	57.8 - 92.9	81.25	71.0 - 89.1	4.22	2.6 - 7.0	0.26	0.1 - 0.6

C

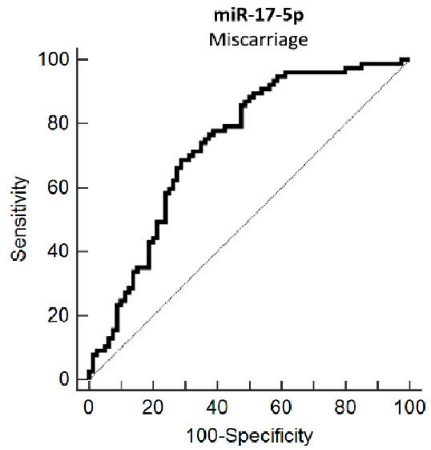

Area under the ROC curve (AUC)	0.736
Standard Error	0.0403
95% Confidence interval	0.660 to 0.803
Significance level P (Area=0.5)	<0.0001

Estimated sensitivity at fixed specificity			
Specificity	Sensitivity	95% CI	Criterion
90.00	24.68	6.49 to 41.56	>3.458809

Criterion	Sensitivity	95% CI	Specificity	95% CI	+LR	95% CI	-LR	95% CI
>1.780378	68.83	57.3 - 78.9	71.25	60.0 - 80.8	2.39	1.6 - 3.5	0.44	0.3 - 0.6

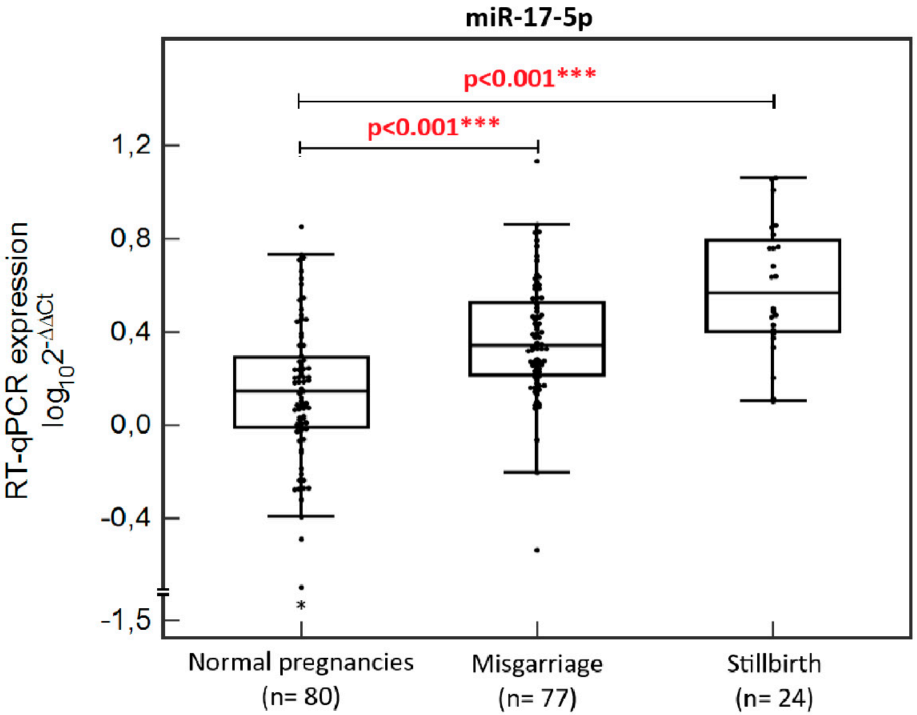

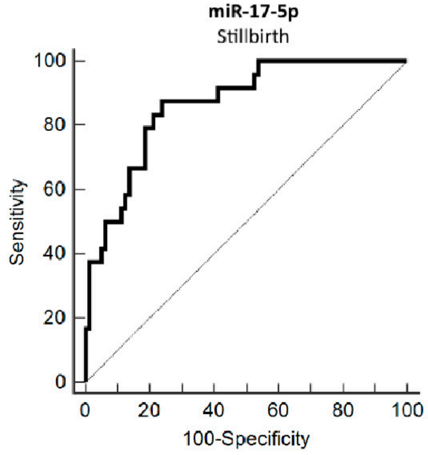

Area under the ROC curve (AUC)	0.865
Standard Error	0.0391
95% Confidence interval	0.784 to 0.924
Significance level P (Area=0.5)	<0.0001

Estimated sensitivity at fixed specificity			
Specificity	Sensitivity	95% CI	Criterion
90.00	50.00	20.83 to 70.83	>3.458809

Criterion	Sensitivity	95% CI	Specificity	95% CI	+LR	95% CI	-LR	95% CI
>1.978627	87.50	67.6 - 97.3	76.25	65.4 - 85.1	3.68	2.4 - 5.6	0.16	0.06 - 0.5

D

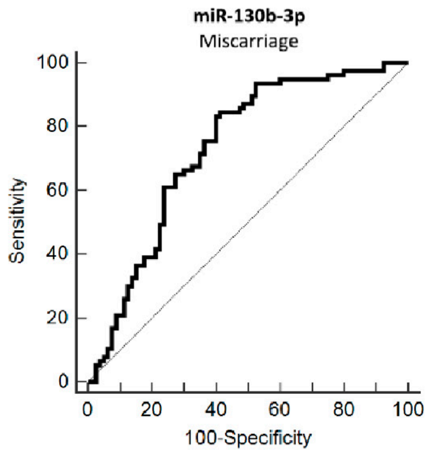

Area under the ROC curve (AUC)	0.731
Standard Error	0.0409
95% Confidence interval	0.654 to 0.798
Significance level P (Area=0.5)	<0.0001

Estimated sensitivity at fixed specificity			
Specificity	Sensitivity	95% CI	Criterion
90.00	20.78	3.90 to 40.26	≤0.235772

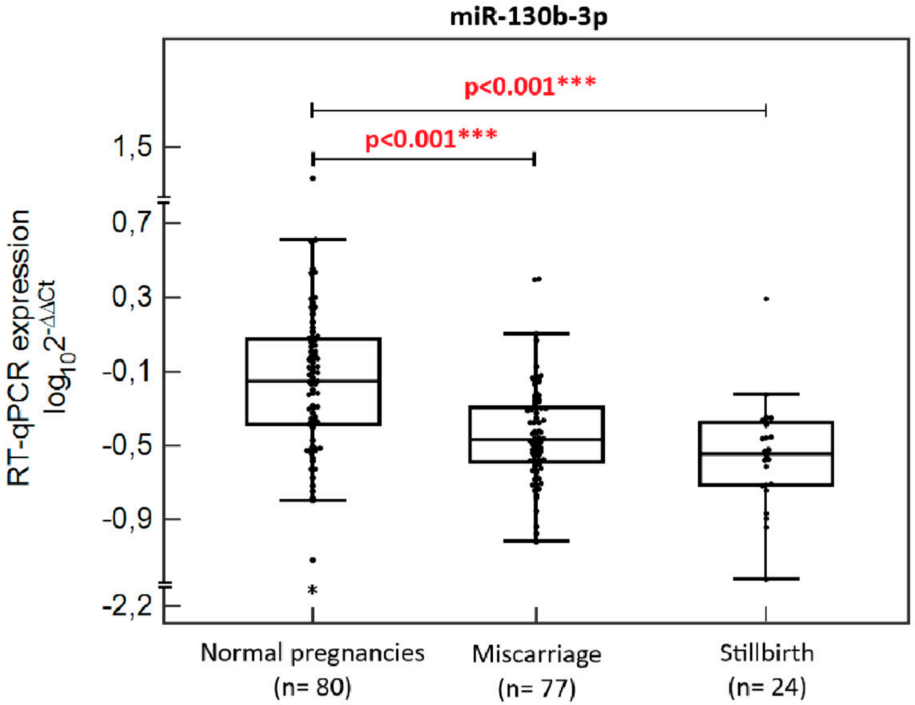

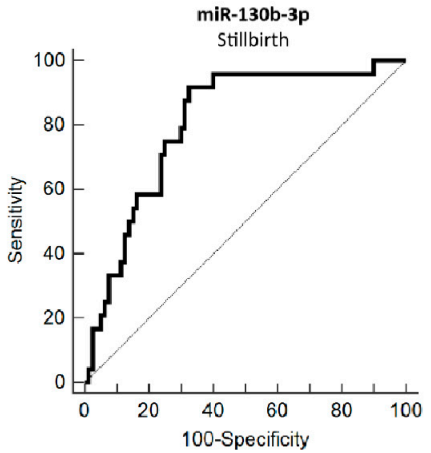

Area under the ROC curve (AUC)	0.805
Standard Error	0.0484
95% Confidence interval	0.716 to 0.876
Significance level P (Area=0.5)	<0.0001

Estimated sensitivity at fixed specificity			
Specificity	Sensitivity	95% CI	Criterion
90.00	33.33	8.33 to 62.50	≤0.235772

Criterion	Sensitivity	95% CI	Specificity	95% CI	+LR	95% CI	-LR	95% CI
≤0.604588314	84.42	74.4 - 91.7	58.75	47.2 - 69.6	2.05	1.5 - 2.7	0.27	0.2 - 0.5

Criterion	Sensitivity	95% CI	Specificity	95% CI	+LR	95% CI	-LR	95% CI
≤0.44464638	91.67	73.0 - 99.0	67.50	56.1 - 77.6	2.82	2.0 - 4.0	0.12	0.03 - 0.5

E

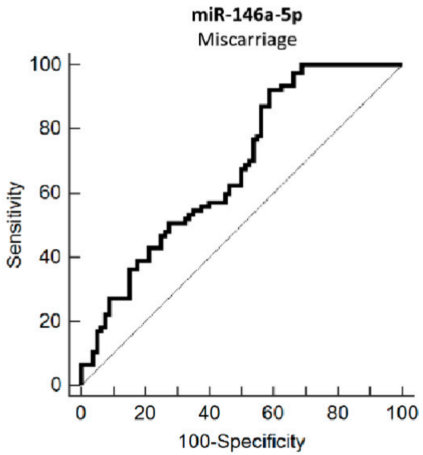

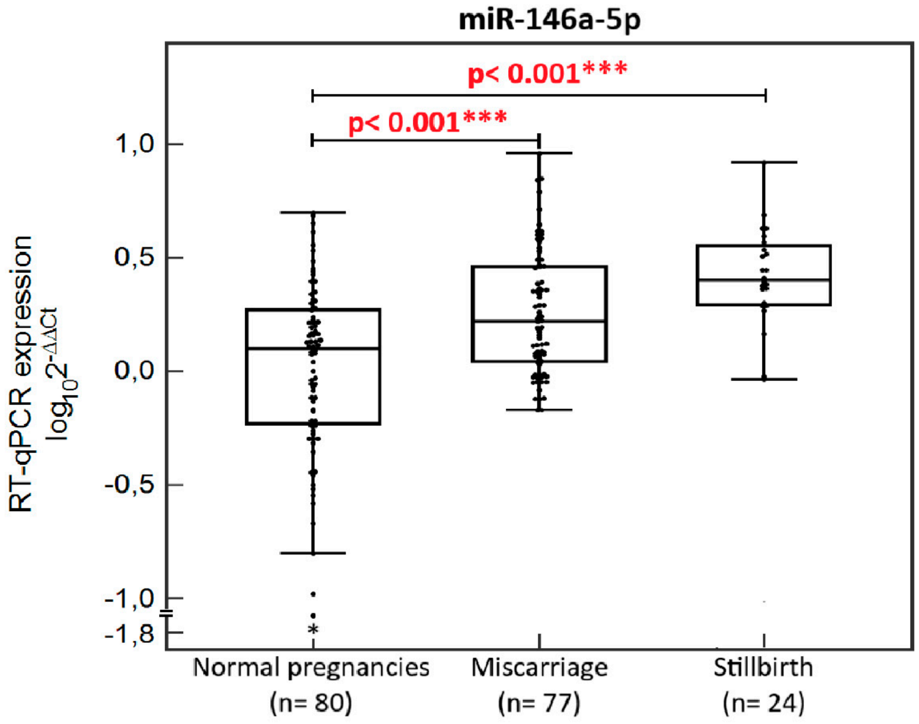

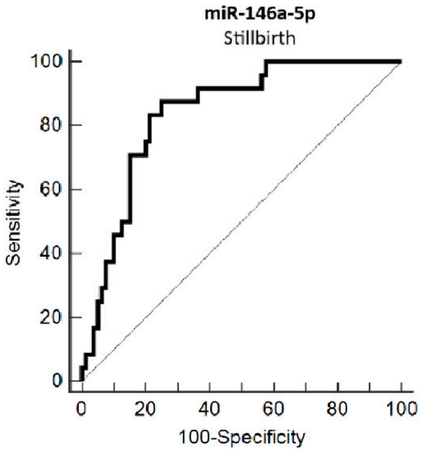

Area under the ROC curve (AUC)	0.678
Standard Error	0.0425
95% Confidence interval	0.599 to 0.751
Significance level P (Area=0.5)	<0.0001

Estimated sensitivity at fixed specificity			
Specificity	Sensitivity	95% CI	Criterion
90.00	27.27	12.99 to 45.45	>2.744534

Criterion	Sensitivity	95% CI	Specificity	95% CI	+LR	95% CI	-LR	95% CI
>0.879161	92.21	83.8 - 97.1	41.25	30.4 - 52.8	1.57	1.3 - 1.9	0.19	0.08 - 0.4

Area under the ROC curve (AUC)	0.840
Standard Error	0.0416
95% Confidence interval	0.755 to 0.904
Significance level P (Area=0.5)	<0.0001

Estimated sensitivity at fixed specificity			
Specificity	Sensitivity	95% CI	Criterion
90.00	45.83	16.67 to 83.33	>2.744534

Criterion	Sensitivity	95% CI	Specificity	95% CI	+LR	95% CI	-LR	95% CI
>1.732512	87.50	67.6 - 97.3	75.00	64.1 - 84.0	3.50	2.3 - 5.3	0.17	0.06 - 0.5

F

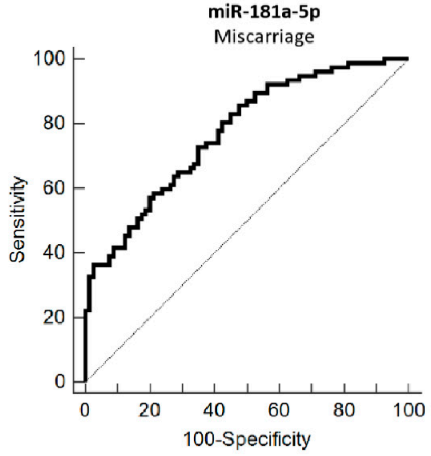

Area under the ROC curve (AUC)	0.772
Standard Error	0.0365
95% Confidence interval	0.699 to 0.835
Significance level P (Area=0.5)	<0.0001

Estimated sensitivity at fixed specificity			
Specificity	Sensitivity	95% CI	Criterion
90.00	41.56	28.57 to 55.84	>0.521629

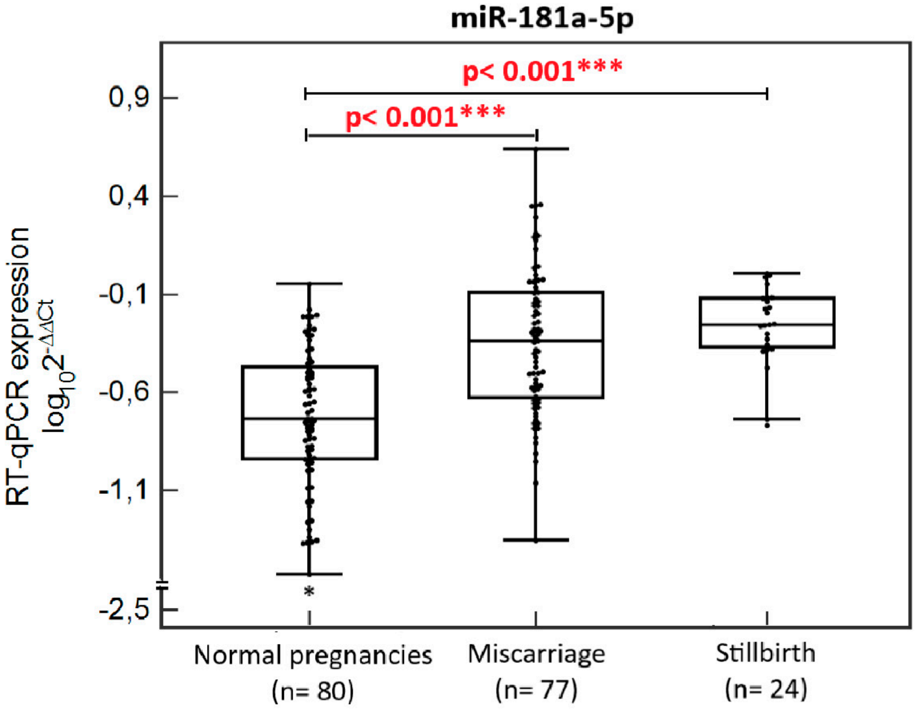

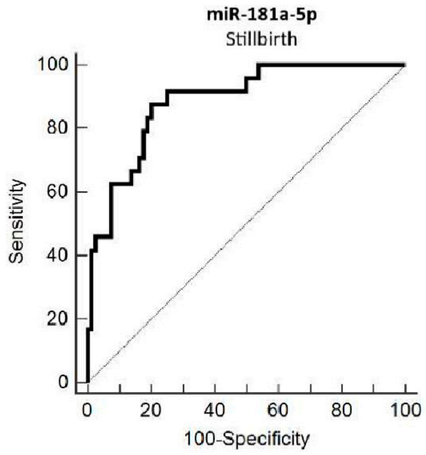

Area under the ROC curve (AUC)	0.886
Standard Error	0.0359
95% Confidence interval	0.809 to 0.940
Significance level P (Area=0.5)	<0.0001

Estimated sensitivity at fixed specificity			
Specificity	Sensitivity	95% CI	Criterion
90.00	62.50	37.50 to 83.33	>0.521629

Criterion	Sensitivity	95% CI	Specificity	95% CI	+LR	95% CI	-LR	95% CI
>0.183648	85.71	75.9 - 92.6	52.50	41.0 - 63.8	1.80	1.4 - 2.3	0.27	0.2 - 0.5

Criterion	Sensitivity	95% CI	Specificity	95% CI	+LR	95% CI	-LR	95% CI
>0.368016	87.50	67.6 - 97.3	80.00	69.6 - 88.1	4.38	2.8 - 7.0	0.16	0.05 - 0.5

G

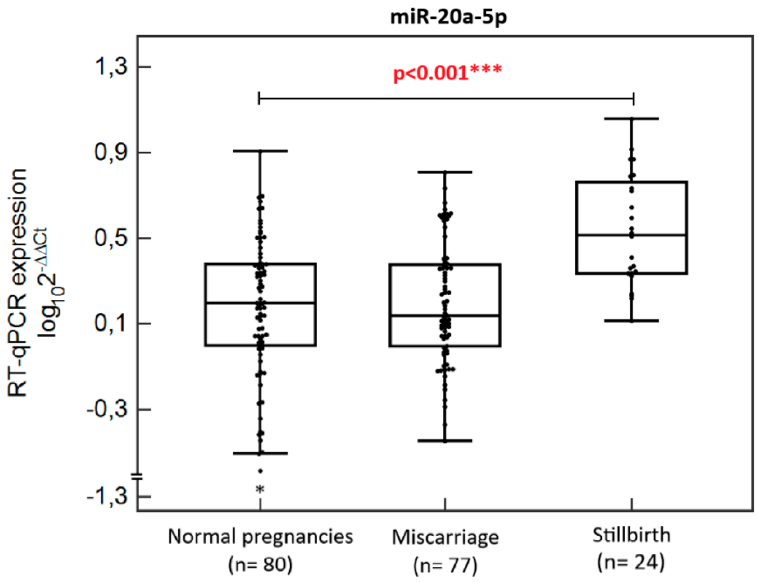

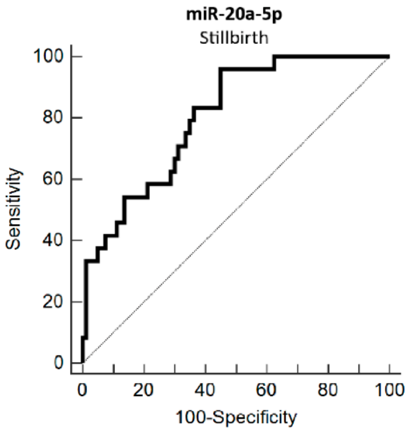

Area under the ROC curve (AUC)	0.803
Standard Error	0.0467
95% Confidence interval	0.714 to 0.875
Significance level P (Area=0.5)	<0.0001

Estimated sensitivity at fixed specificity			
Specificity	Sensitivity	95% CI	Criterion
90.00	41.67	20.83 to 62.50	>3.587078

Criterion	Sensitivity	95% CI	Specificity	95% CI	+LR	95% CI	-LR	95% CI
>1.6574	95.83	78.9 - 99.9	55.00	43.5 - 66.2	2.13	1.6 - 2.8	0.076	0.01 - 0.5

H

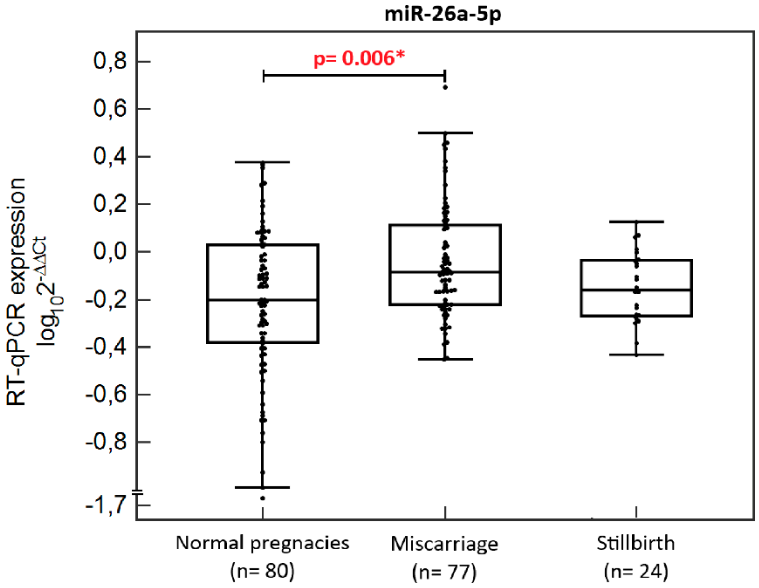

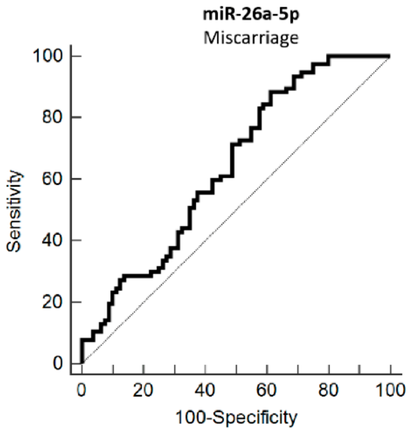

Area under the ROC curve (AUC)	0.640
Standard Error	0.0441
95% Confidence interval	0.560 to 0.715
Significance level P (Area=0.5)	0.0015

Estimated sensitivity at fixed specificity			
Specificity	Sensitivity	95% CI	Criterion
90.00	23.38	9.09 to 37.66	>1.350228

Criterion	Sensitivity	95% CI	Specificity	95% CI	+LR	95% CI	-LR	95% CI
>0.525138	88.31	79.0 - 94.5	38.75	28.1 - 50.3	1.44	1.2 - 1.7	0.30	0.2 - 0.6

I

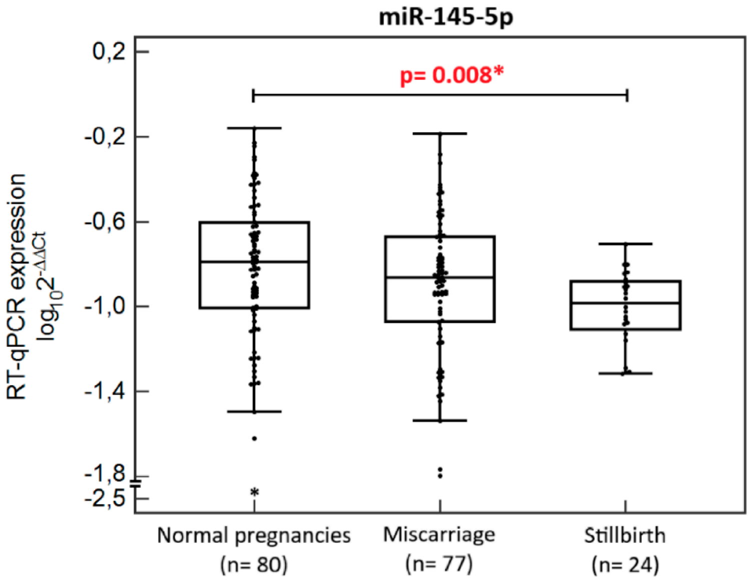

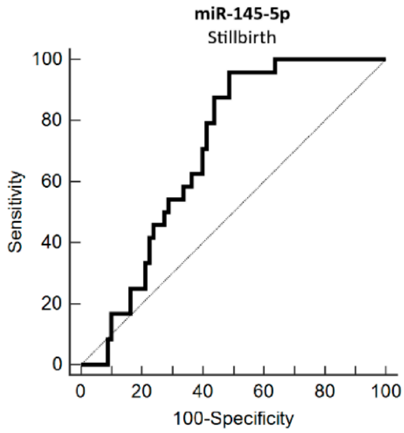

Area under the ROC curve (AUC)	0.701
Standard Error	0.0504
95% Confidence interval	0.603 to 0.786
Significance level P (Area=0.5)	0.0001

Estimated sensitivity at fixed specificity

Specificity	Sensitivity	95% CI	Criterion
90.00	16.67	0.00 to 41.67	≤0.051862838

Criterion	Sensitivity	95% CI	Specificity	95% CI	+LR	95% CI	-LR	95% CI
≤0.15890505	95.83	78.9 - 99.9	51.25	39.8 - 62.6	1.97	1.5 - 2.5	0.081	0.01 - 0.6

J

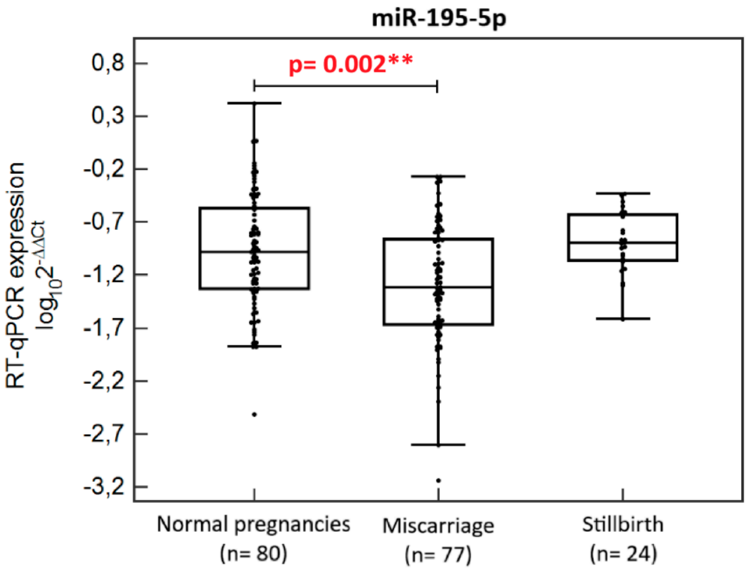

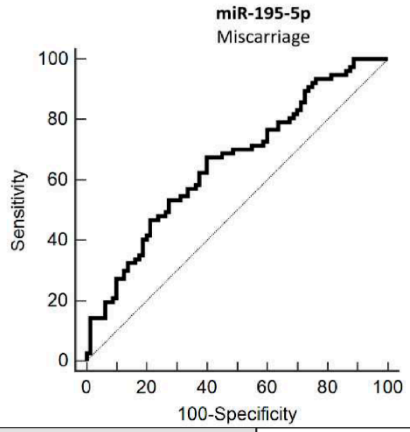

Area under the ROC curve (AUC)	0.657
Standard Error	0.0434
95% Confidence interval	0.577 to 0.731
Significance level P (Area=0.5)	0.0003

Estimated sensitivity at fixed specificity

Specificity	Sensitivity	95% CI	Criterion
90.00	27.27	12.99 to 45.63	≤0.022487627

Criterion	Sensitivity	95% CI	Specificity	95% CI	+LR	95% CI	-LR	95% CI
≤0.082537076	67.53	55.9 - 77.8	60.00	48.4 - 70.8	1.69	1.2 - 2.3	0.54	0.4 - 0.8

K

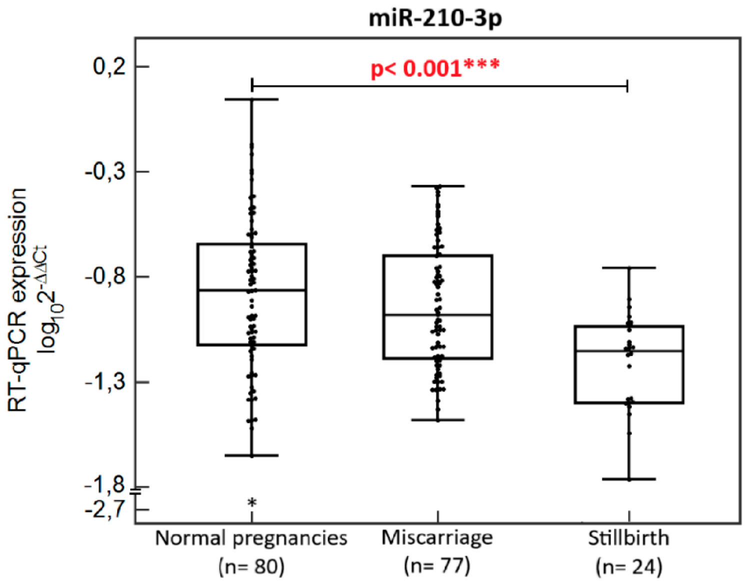

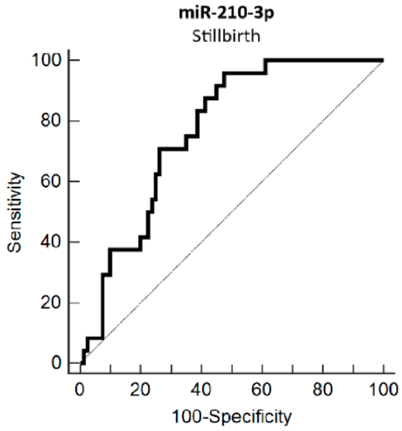

Area under the ROC curve (AUC)	0.767
Standard Error	0.0472
95% Confidence interval	0.674 to 0.844
Significance level P (Area=0.5)	<0.0001

Estimated sensitivity at fixed specificity			
Specificity	Sensitivity	95% CI	Criterion
90.00	37.50	12.50 to 66.67	≤0.0418995

Criterion	Sensitivity	95% CI	Specificity	95% CI	+LR	95% CI	-LR	95% CI
≤0.124362787	95.83	78.9 - 99.9	52.50	41.0 - 63.8	2.02	1.6 - 2.6	0.079	0.01 - 0.5

L

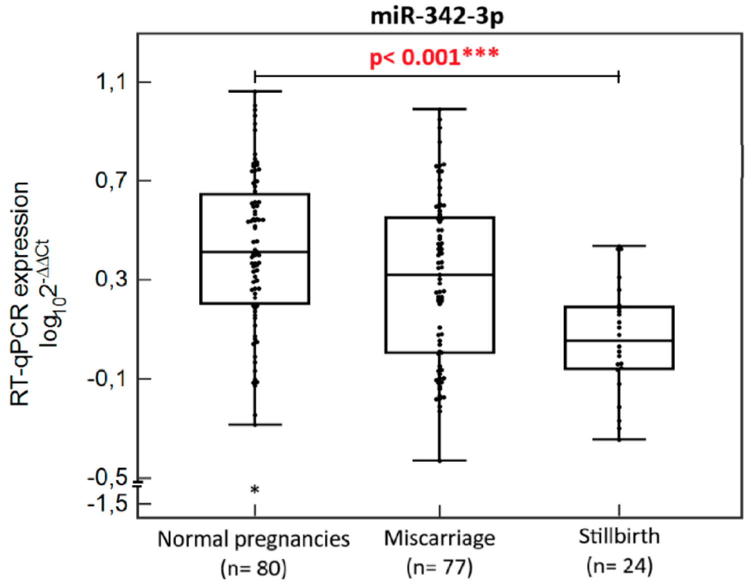

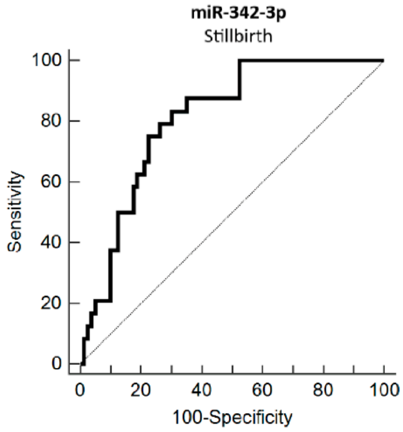

Area under the ROC curve (AUC)	0.808
Standard Error	0.0441
95% Confidence interval	0.719 to 0.879
Significance level P (Area=0.5)	<0.0001

Estimated sensitivity at fixed specificity			
Specificity	Sensitivity	95% CI	Criterion
90.00	37.50	12.50 to 75.00	≤0.909153316

Criterion	Sensitivity	95% CI	Specificity	95% CI	+LR	95% CI	-LR	95% CI
≤1.81094546	83.33	62.6 - 95.3	70.00	58.7 - 79.7	2.78	1.9 - 4.1	0.24	0.10 - 0.6

M

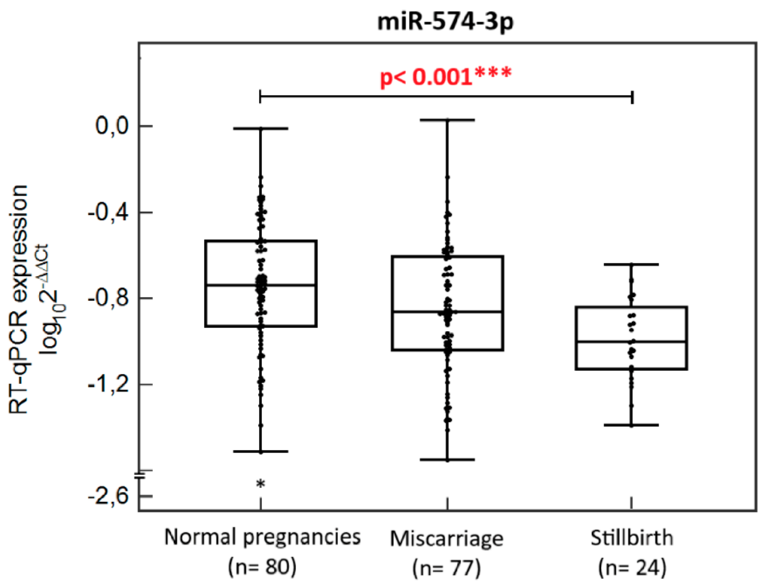

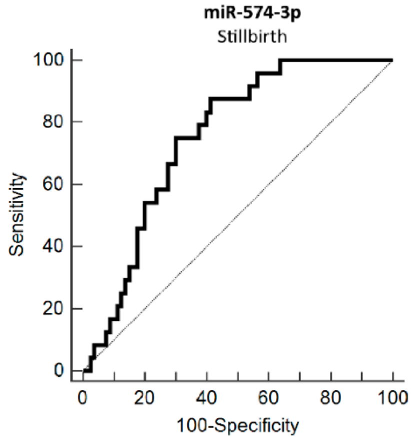

Area under the ROC curve (AUC)	0.751
Standard Error	0.0488
95% Confidence interval	0.656 to 0.830
Significance level P (Area=0.5)	<0.0001

Estimated sensitivity at fixed specificity			
Specificity	Sensitivity	95% CI	Criterion
90.00	16.67	0.00 to 50.00	≤0.06522

Criterion	Sensitivity	95% CI	Specificity	95% CI	+LR	95% CI	-LR	95% CI
≤0.16346195	87.50	67.6 - 97.3	58.75	47.2 - 69.6	2.12	1.6 - 2.9	0.21	0.07 - 0.6

Supplementary Figure S2. Comparison of miRNAs gene expression in peripheral blood leukocytes in early stages of gestation between pregnancies with normal course and those with subsequent miscarriage or stillbirth. Significant dysregulation (after the Benjamini-Hochberg correction) of miR-1-3p (**A**), miR-16-5p (**B**), miR-17-5p (**C**), miR-130b-3p (**D**), miR-146a-5p (**E**), and miR-181a-5p (**F**) in both groups, pregnancies with subsequent miscarriage or stillbirth; significant dysregulation (after the Benjamini-Hochberg correction) of miR-20a-5p (**G**), miR-145-5p (**I**), miR-210-3p (**K**), miR-342-3p (**L**), and miR-574-3p (M) in the group of pregnancies with subsequent stillbirth and miR-26a-5p (**H**) and miR-195-5p (**J**) in the group of pregnancies with subsequent miscarriage only.

(results after the Benjamini-Hochberg correction are marked by * for α=0.05, ** for α=0.01, and *** for α=0.001)
