# Supplementary material for: First-Trimester Screening for Miscarriage or Stillbirth—Prediction Model Based on MicroRNA Biomarkers
Source: Int J Mol Sci. 2023 Jun 14;24(12):10137. doi: 10.3390/ijms241210137 (PMC10299132; doi:10.3390/ijms241210137)
Supplement: Supplementary file 1 [file ijms-24-10137-s001.zip › Supplementary Figure S3.pdf]

Supplementary Figure S3.

A

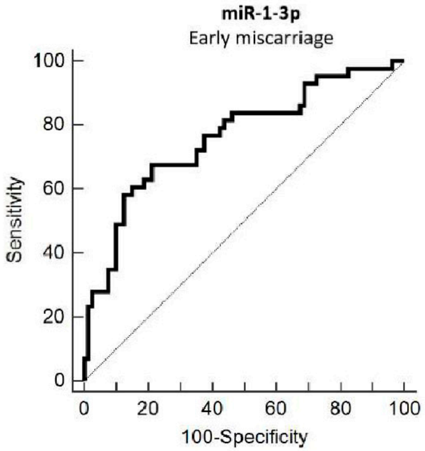

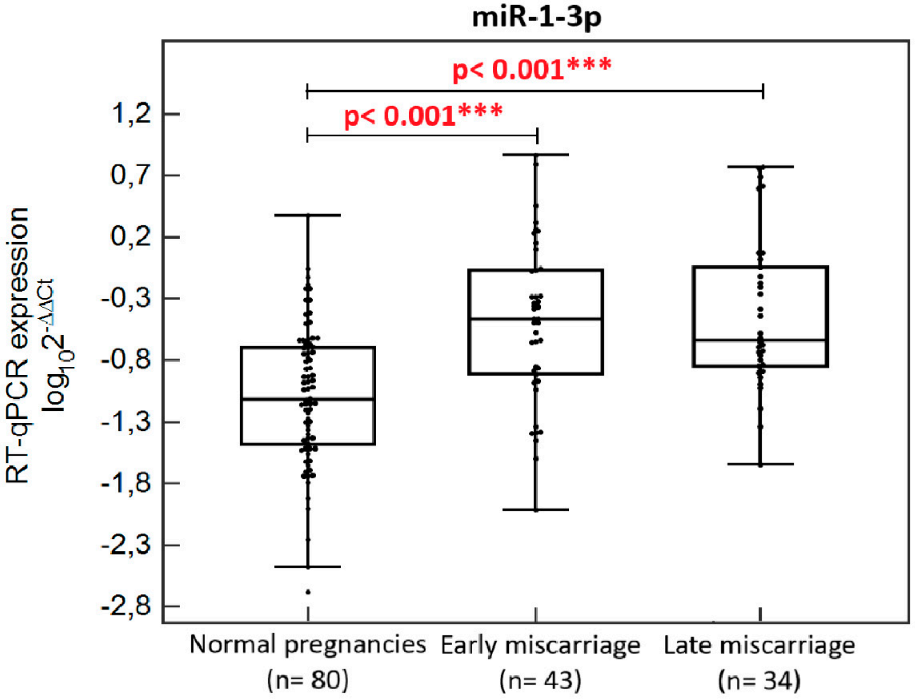

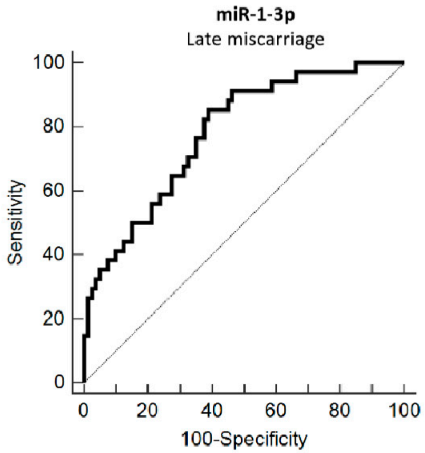

Area under the ROC curve (AUC)	0.762
Standard Error	0.0470
95% Confidence interval	0.676 to 0.834
Significance level P (Area=0.5)	<0.0001

Estimated sensitivity at fixed specificity			
Specificity	Sensitivity	95% CI	Criterion
90.00	48.84	23.26 to 72.09	>0.377841

Area under the ROC curve (AUC)	0.781
Standard Error	0.0456
95% Confidence interval	0.693 to 0.853
Significance level P (Area=0.5)	<0.0001

Estimated sensitivity at fixed specificity			
Specificity	Sensitivity	95% CI	Criterion
90.00	41.18	23.53 to 61.76	>0.377841

Criterion	Sensitivity	95% CI	Specificity	95% CI	+LR	95% CI	-LR	95% CI
>0.210938	67.44	51.5 - 80.9	78.75	68.2 - 87.1	3.17	2.0 - 5.1	0.41	0.3 - 0.6

Criterion	Sensitivity	95% CI	Specificity	95% CI	+LR	95% CI	-LR	95% CI
>0.115119	85.29	68.9 - 95.0	61.25	49.7 - 71.9	2.20	1.6 - 3.0	0.24	0.1 - 0.5

B

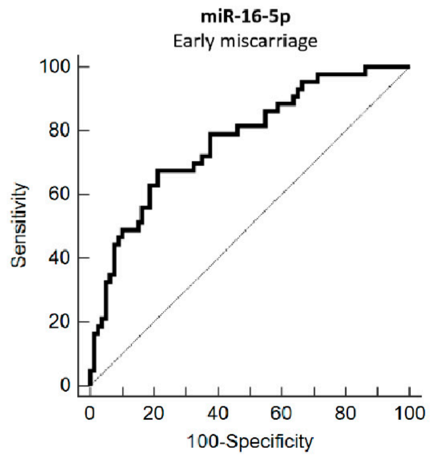

Area under the ROC curve (AUC)	0.771
Standard Erro	0.0445
95% Confidence interval	0.686 to 0.842
Significance level P (Area=0.5)	<0.0001

Estimated sensitivity at fixed specificity			
Specificity	Sensitivity	95% CI	Criterion
90.00	48.84	27.91 to 69.77	>3.02212

Criterion	Sensitivity	95% CI	Specificity	95% CI	+LR	95% CI	-LR	95% CI
>2.085522	67.44	51.5 - 80.9	78.75	68.2 - 87.1	3.17	2.0 - 5.1	0.41	0.3 - 0.6

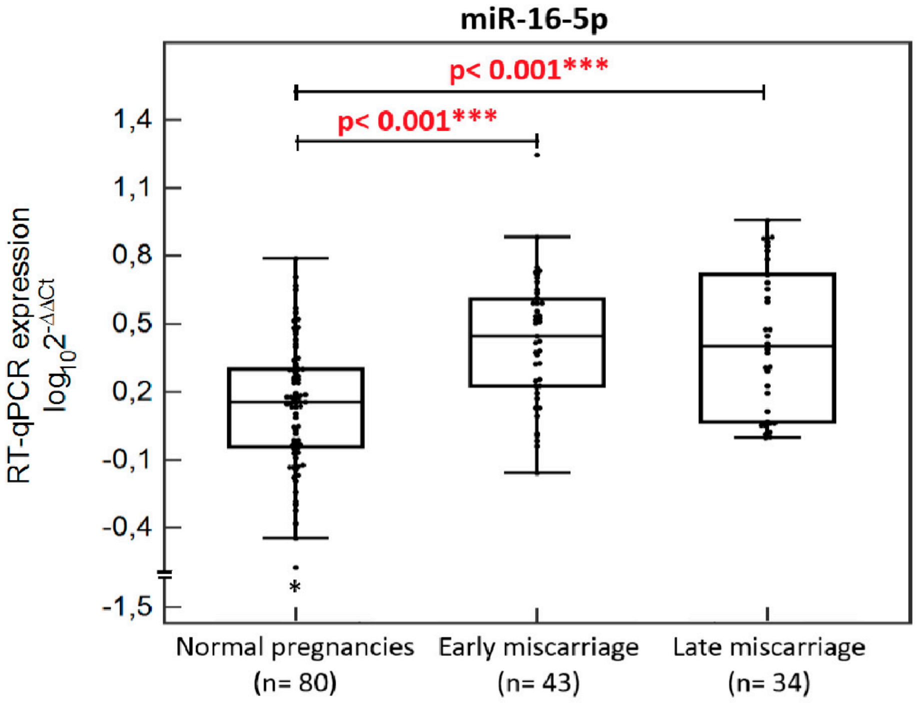

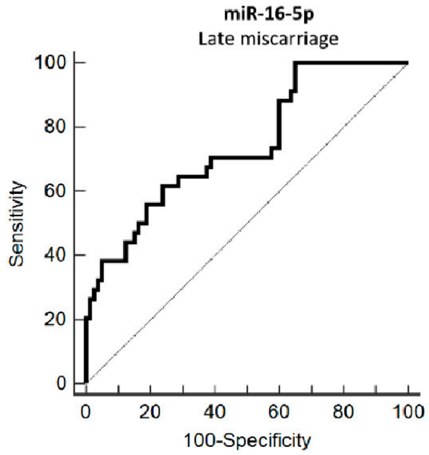

Area under the ROC curve (AUC)	0.741
Standard Error	0.0510
95% Confidence interval	0.650 to 0.818
Significance level P (Area=0.5)	<0.0001

Estimated sensitivity at fixed specificity			
Specificity	Sensitivity	95% CI	Criterion
90.00	38.24	20.59 to 55.88	>3.02212

Criterion	Sensitivity	95% CI	Specificity	95% CI	+LR	95% CI	-LR	95% CI
>1.983489	61.76	43.6 - 77.8	76.25	65.4 - 85.1	2.60	1.6 - 4.2	0.50	0.3 - 0.8

C

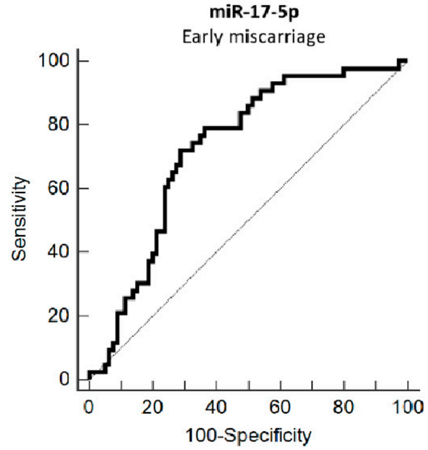

Area under the ROC curve (AUC)	0.726
Standard Error	0.0466
95% Confidence interval	0.638 to 0.802
Significance level P (Area=0.5)	<0.0001

Estimated sensitivity at fixed specificity

Specificity	Sensitivity	95% CI	Criterion
90.00	20.93	2.33 to 41.86	>3.458809

Criterion	Sensitivity	95% CI	Specificity	95% CI	+LR	95% CI	-LR	95% CI
>1.780378	72.09	56.3 - 84.7	71.25	60.0 - 80.8	2.51	1.7 - 3.7	0.39	0.2 - 0.6

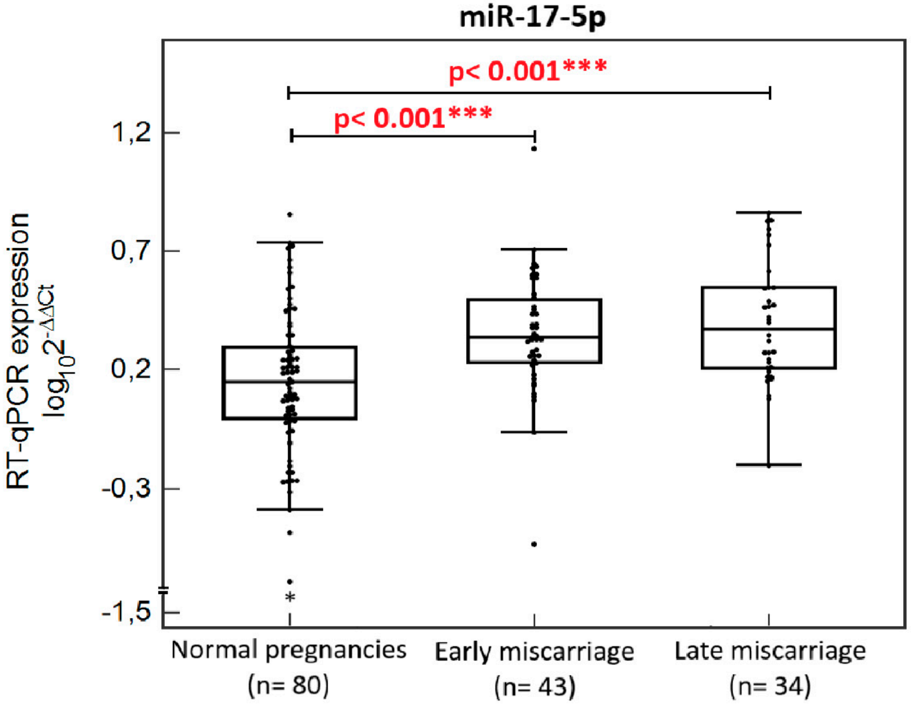

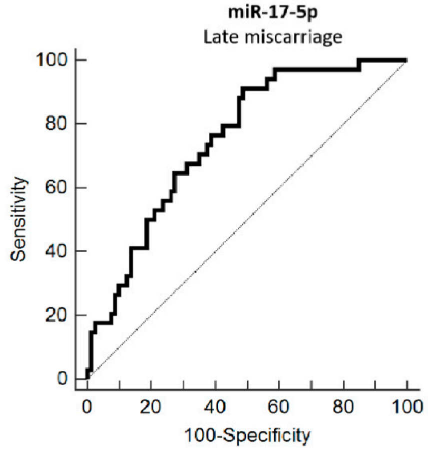

Area under the ROC curve (AUC)	0.749
Standard Error	0.0469
95% Confidence interval	0.659 to 0.825
Significance level P (Area=0.5)	<0.0001

Estimated sensitivity at fixed specificity

Specificity	Sensitivity	95% CI	Criterion
90.00	29.41	8.82 to 52.94	>3.458809

Criterion	Sensitivity	95% CI	Specificity	95% CI	+LR	95% CI	-LR	95% CI
>1.396173	91.18	76.3 - 98.1	51.25	39.8 - 62.6	1.87	1.5 - 2.4	0.17	0.06 - 0.5

D

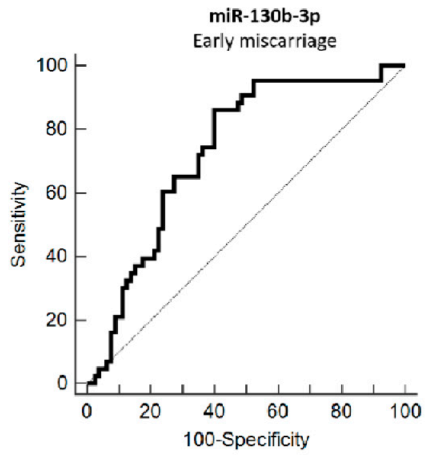

Area under the ROC curve (AUC)	0.732
Standard Error	0.0462
95% Confidence interval	0.644 to 0.808
Significance level P (Area=0.5)	<0.0001

Estimated sensitivity at fixed specificity			
Specificity	Sensitivity	95% CI	Criterion
90.00	20.93	0.00 to 44.19	≤0.235772

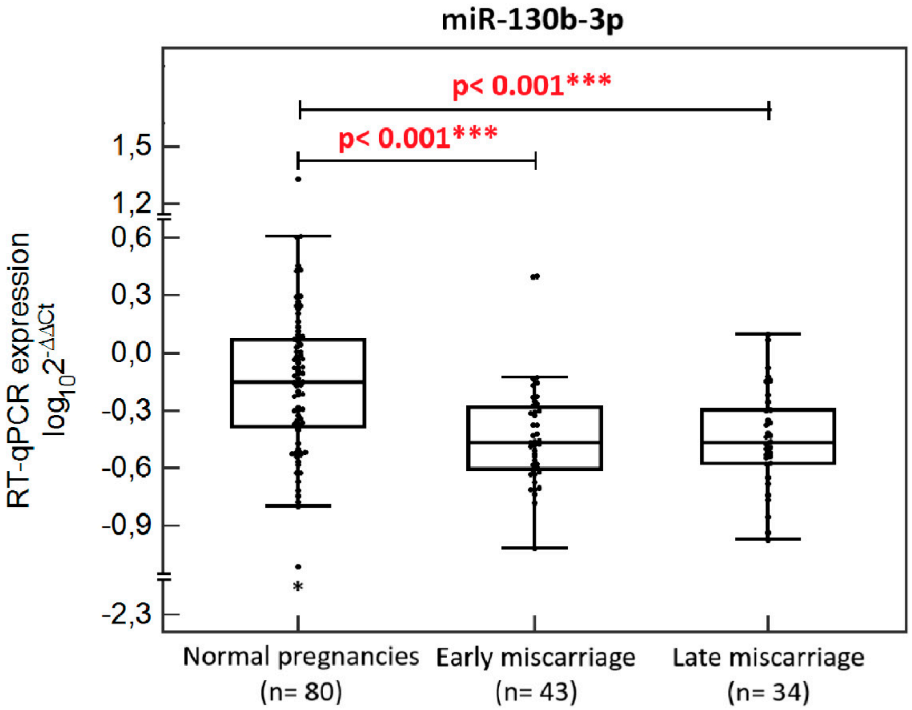

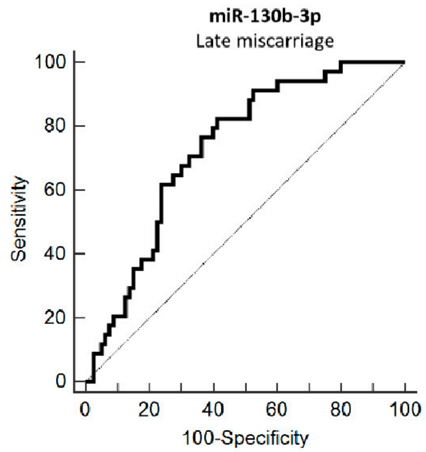

Area under the ROC curve (AUC)	0.730
Standard Error	0.0481
95% Confidence interval	0.639 to 0.809
Significance level P (Area=0.5)	<0.0001

Estimated sensitivity at fixed specificity			
Specificity	Sensitivity	95% CI	Criterion
90.00	20.59	5.88 to 44.12	≤0.235772

Criterion	Sensitivity	95% CI	Specificity	95% CI	+LR	95% CI	-LR	95% CI
≤0.516907	74.42	58.8 - 86.5	60.00	48.4 - 70.8	1.86	1.4 - 2.6	0.43	0.2 - 0.7

Criterion	Sensitivity	95% CI	Specificity	95% CI	+LR	95% CI	-LR	95% CI
≤0.604588314	82.35	65.5 - 93.2	58.75	47.2 - 69.6	2.00	1.5 - 2.7	0.30	0.1 - 0.6

E

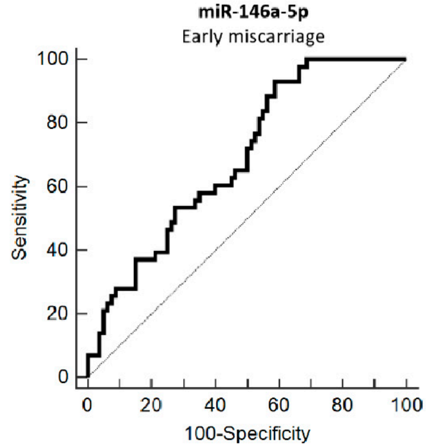

Area under the ROC curve (AUC)	0.690
Standard Error	0.0477
95% Confidence interval	0.600 to 0.770
Significance level P (Area=0.5)	0.0001

Estimated sensitivity at fixed specificity

Specificity	Sensitivity	95% CI	Criterion
90.00	27.91	13.95 to 47.28	>2.744534

Criterion	Sensitivity	95% CI	Specificity	95% CI	+LR	95% CI	-LR	95% CI
>0.879161	93.02	80.9 - 98.5	41.25	30.4 - 52.8	1.58	1.3 - 1.9	0.17	0.06 - 0.5

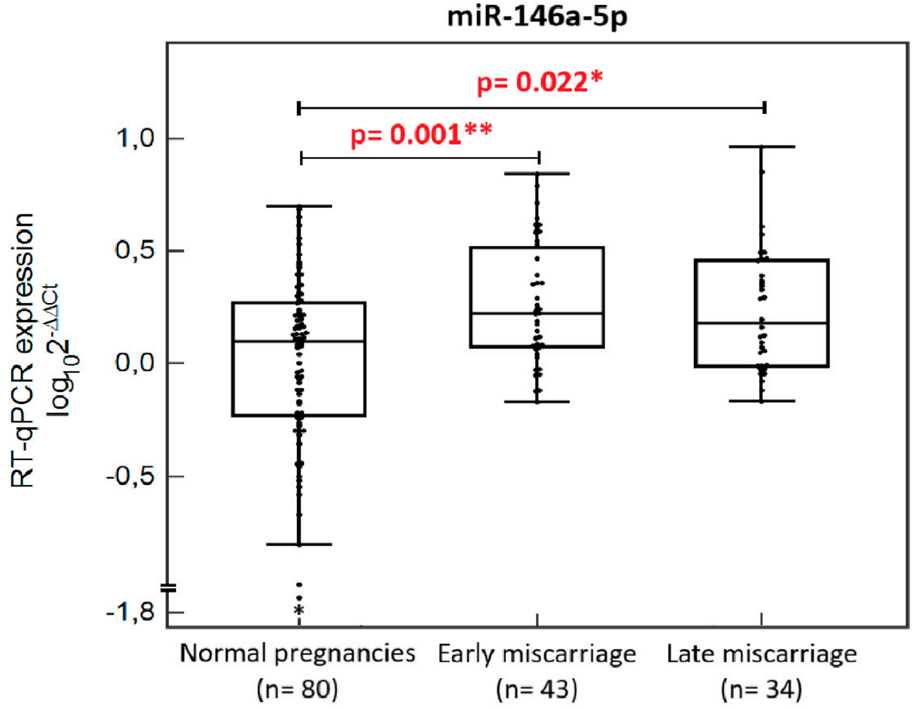

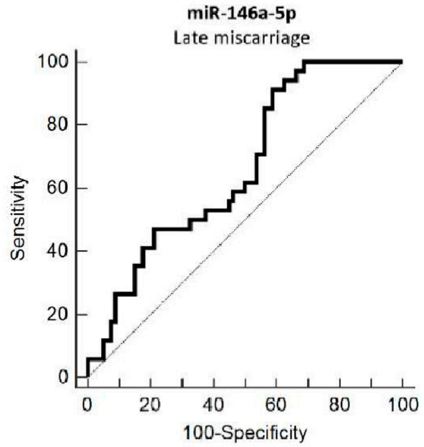

Area under the ROC curve (AUC)	0.664
Standard Error	0.0527
95% Confidence interval	0.569 to 0.750
Significance level P (Area=0.5)	0.0019

Estimated sensitivity at fixed specificity

Specificity	Sensitivity	95% CI	Criterion
90.00	26.47	8.82 to 52.94	>2.744534

Criterion	Sensitivity	95% CI	Specificity	95% CI	+LR	95% CI	-LR	95% CI
>0.879161	91.18	76.3 - 98.1	41.25	30.4 - 52.8	1.55	1.3 - 1.9	0.21	0.07 - 0.7

F

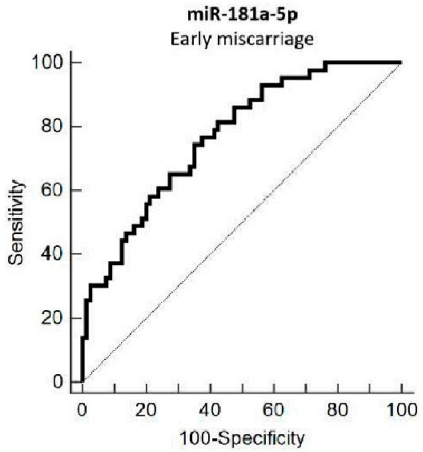

Area under the ROC curve (AUC)	0.769
Standard Error	0.0429
95% Confidence interval	0.685 to 0.840
Significance level P (Area=0.5)	<0.0001

Estimated sensitivity at fixed specificity			
Specificity	Sensitivity	95% CI	Criterion
90.00	37.21	20.25 to 57.93	>0.521629

Criterion	Sensitivity	95% CI	Specificity	95% CI	+LR	95% CI	-LR	95% CI
>0.261811	74.42	58.8 - 86.5	65.00	53.5 - 75.3	2.13	1.5 - 3.0	0.39	0.2 - 0.7

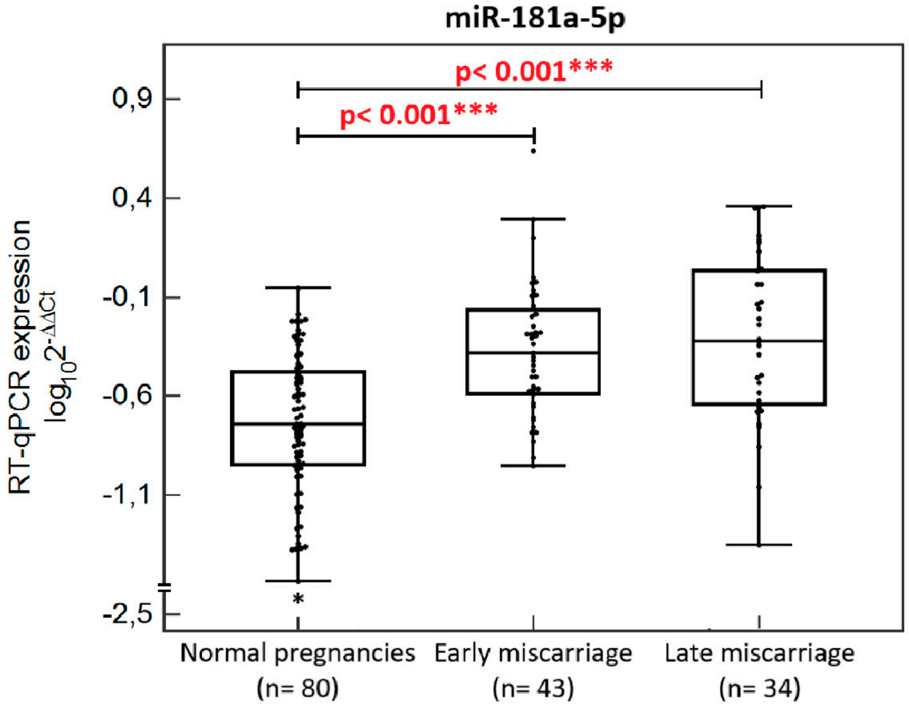

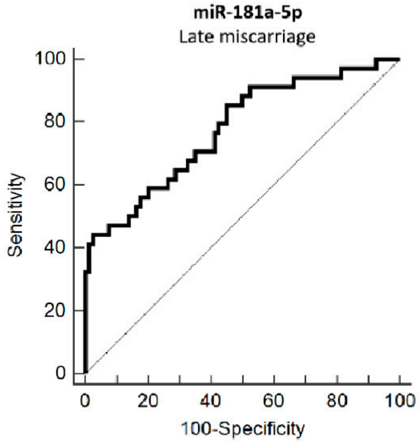

Area under the ROC curve (AUC)	0.776
Standard Error	0.0495
95% Confidence interval	0.688 to 0.849
Significance level P (Area=0.5)	<0.0001

Estimated sensitivity at fixed specificity			
Specificity	Sensitivity	95% CI	Criterion
90.00	47.06	29.41 to 64.71	>0.521629

Criterion	Sensitivity	95% CI	Specificity	95% CI	+LR	95% CI	-LR	95% CI
>0.616153	44.12	27.2 - 62.1	97.50	91.3 - 99.7	17.65	4.3 - 73.0	0.57	0.4 - 0.8

G

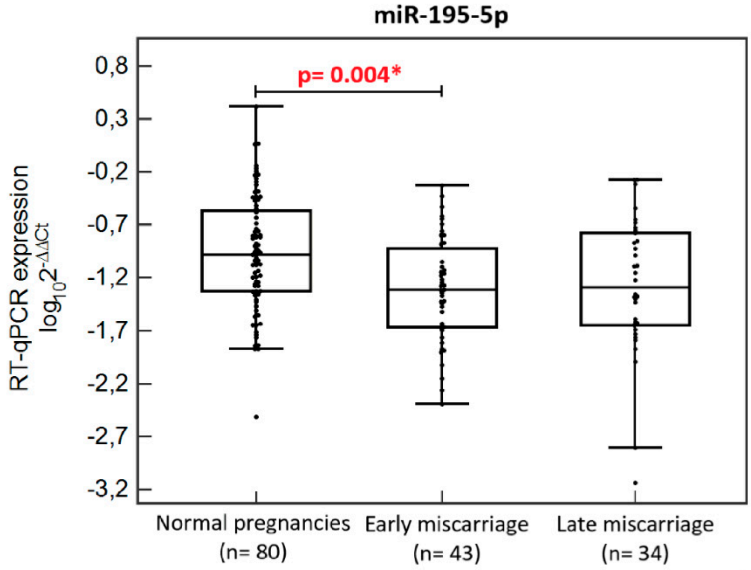

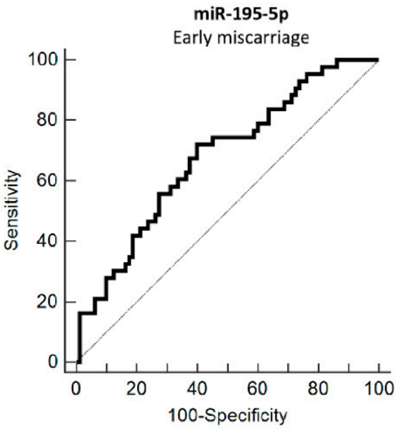

Area under the ROC curve (AUC)	0.678
Standard Error	0.0501
95% Confidence interval	0.588 to 0.759
Significance level P (Area=0.5)	0.0004

Estimated sensitivity at fixed specificity			
Specificity	Sensitivity	95% CI	Criterion
90.00	27.91	13.95 to 51.16	≤0.021711216

Criterion	Sensitivity	95% CI	Specificity	95% CI	+LR	95% CI	-LR	95% CI
≤0.07941059	72.09	56.3 - 84.7	60.00	48.4 - 70.8	1.80	1.3 - 2.5	0.47	0.3 - 0.8

Supplementary Figure S3. Increased levels of miR-1-3p (**A**), miR-16-5p (**B**), miR-17-5p (**C**), miR-146a-5p (**E**), and miR-181a-5p (**F**), and decreased levels of miR-130b-3p (**D**) in early stages of gestation in pregnancies with an early miscarriage occurring before 13 gestational weeks and a late miscarriage occurring within 13 and 20 gestational weeks; decreased levels of miR-195-5p (**G**) in pregnancies with an early miscarriage only.
(results after the Benjamini-Hochberg correction are marked by * for α=0.05, ** for α=0.01, and *** for α=0.001)
